# Patient Derived Xenografts (PDX) Models as an Avatar to Assess Personalized Therapy Options in Uveal Melanoma: A Feasibility Study

**DOI:** 10.3390/curroncol30100657

**Published:** 2023-10-11

**Authors:** Fariba Nemati, Leanne de Koning, David Gentien, Franck Assayag, Emilie Henry, Khadija Ait Rais, Gaelle Pierron, Odette Mariani, Michèle Nijnikoff, Gabriel Champenois, André Nicolas, Didier Meseure, Sophie Gardrat, Nicolas Servant, Philippe Hupé, Maud Kamal, Christophe Le Tourneau, Sophie Piperno-Neumann, Manuel Rodrigues, Sergio Roman-Roman, Didier Decaudin, Pascale Mariani, Nathalie Cassoux

**Affiliations:** 1 Laboratory of Preclinical Investigation, Translational Research Department, Institut Curie, PSL University Paris, 26 rue d’Ulm, CEDEX 05, 75248 Paris, France; 2 Translational Research Department, Institut Curie, PSL University Paris, 75248 Paris, France; leanne.de-koning@curie.fr (L.d.K.);; 3 Genomics Platform, Translational Research Department, Institut Curie, PSL Research University, 75248 Paris, France; 4 Department of Genetics, Institut Curie, PSL Research University, 75248 Paris, France; 5 Biological Resource Center, Department of Pathology, Institut Curie, PSL Research University, 75248 Paris, France; 6 Department of Biopathology, Institut Curie, PSL Research University, 75248 Paris, France; 7 Institut Curie, INSERM U900, CBIO-Centre for Computational Biology, Mines Paris Tech, PSL-Research University, 75248 Paris, France; 8 Department of Drug Development and Innovation (D3i), Institut Curie, 75248 Paris, France; 9 INSERM U900 Research Unit, Institut Curie, 92064 Saint-Cloud, France; 10 Paris-Saclay University, 75248 Paris, France; 11 Department of Medical Oncology, Institut Curie, PSL Research University, 75248 Paris, France; 12 Department of Surgical Oncology, Institut Curie, PSL Research University, 75248 Paris, France; 13 Department of Oncological Ophthalmology, Institut Curie, Université Paris Cité, 75248 Paris, France

**Keywords:** patient-derived xenografts (PDXs), uveal melanoma, avatar

## Abstract

Uveal melanoma is the most common primary intraocular malignancy in adults. Up to 50% of UM patients develop metastatic disease, usually in the liver. When metastatic, the prognosis is poor, and few treatment options exist. Here, we investigated the feasibility of establishing patient-derived xenografts (PDXs) from a patient’s tumor in order to screen for therapies that the patient could benefit from. Samples obtained from 29 primary tumors and liver metastases of uveal melanoma were grafted into SCID mice. PDX models were successfully established for 35% of primary patient tumors and 67% of liver metastases. The tumor take rate was proportional to the risk of metastases. PDXs showed the same morphology, the same GNAQ/11, BAP1, and SF3B1 mutations, and the same chromosome 3 and 8q status as the corresponding patient samples. Six PDX models were challenged with two compounds for 4 weeks. We show that, for 31% of patients with high or intermediate risk of metastasis, the timing to obtain efficacy results on PDX models derived from their primary tumors was compatible with the selection of the therapy to treat the patient after relapse. PDXs could thus be a valid tool (“avatar”) to select the best personalized therapy for one third of patients that are most at risk of relapse.

## 1. Introduction

Uveal melanoma (UM) is the most common primary intraocular malignancy in adults, with an incidence of 5–8 per million individuals per year in Caucasian populations [1]. Despite successful treatment of the primary tumor by either surgery and/or radiotherapy, up to 50% of UM patients develop metastatic disease, usually in the liver, even several years after the primary treatment [2,3]. The median interval between primary tumor and metastasis was 68 months (range 19–81) in our series [4]. Tools based on gene expression profiling (Decision Dx-UM [5]) or on genetic aberrations [6] allow us to estimate the risk of metastasis and to adapt surveillance. After a diagnosis of liver metastases, the median overall survival is around 15 months [3,7,8], and treatment options are limited at this stage. Although a small fraction (<5%) of patients show an unexpected response to treatment, the short survival time of most patients does not generally allow testing several lines of subsequent treatments. Therefore, tumor models derived from each individual patient would allow us to test different treatment options in parallel and selecting the most efficient one(s) for the patient. Patient-derived xenografts (PDXs) are in vivo models which are based on the graft of human tumor fragments in immunocompromised mice [9,10,11,12]. These xenografts retain the histopathological and genetic features of the original patients’ cancers, making them a valid tool to expand primary tumors, predict cancer response to therapy, and determine new therapeutic biomarkers [13,14,15,16,17,18]. PDX mice models can be used as an avatar of the original tumor to test several drugs or a combination of drugs simultaneously and select the best therapy for their corresponding patients. The therapeutic benefits of avatar mice have been demonstrated by several groups in colon cancer, lung cancer, pancreatic cancer, renal carcinoma, and breast cancer [10,17,18,19,20,21,22,23]. Moreover, PDX models have been used as tools for clinical trials and co-clinical trials [12].

In UM, these avatar models could be of particular interest, since primary tumors and metastases tend to be highly similar, at least from a genomic point of view [24]. These models could thus potentially be developed from the primary tumor material in anticipation of metastatic recurrence and allow for the rapid testing of multiple drugs.

The present study is a proof-of-concept study evaluating the feasibility of avatar models to predict individual therapy responses in UM. We evaluated whether the time required to obtain the PDXs from the primary tumor, and to challenge them with different treatments, is compatible with the short life expectancy of the patients.

## 2. Materials and Methods

### 2.1. Patients and Tumor Samples

A total of 30 patient samples were obtained from surgical left-over. All patients had previously given their informed consent for experimental research on residual tumor tissues. One of the samples was not tumoral and was excluded. Among the remaining 29 samples, 20 were obtained from primary UM and 9 from liver metastases. Table 1 summarizes the characteristics of patients. Twelve samples came from men and seventeen from women. Age ranged from 30 to 83 years old in both men and women. After surgery, some tumor specimens were fixed for further morphological and histological analyses, and others were frozen for further genomic analyses.

### 2.2. Establishment of Uveal Melanoma Xenografts

Fresh tumor samples obtained from pathologists were transplanted into the interscapular fat pad of two to four immunodeficient female SCID mice (Janvier Labs, Paris, France), 5 to 7 weeks old, under total xylazine/ketamine anesthesia (=passage 0). Mice were maintained in specific pathogen-free animal housing (Institut Curie, Paris, France) and regularly observed for tumor growth. Animal care and use for this study were performed following the recommendations of the European Community (2010/63/UE) for the care and use of laboratory animals and under the supervision of authorized investigators (APAFIS#25870-2020060410487032 v1). Mice were weighed and tumors were measured at least once a week. Tumor volumes (V), calculated by measuring two perpendicular diameters with calipers, were calculated according to the formula V = a × b^2^/2, where a and b are the largest and smallest perpendicular tumor diameters. At a volume of ~800 mm^3^, tumors were removed, and specimens were frozen directly in liquid nitrogen for molecular analysis, or in a DMSO-FCS solution for cryopreservation and expansion. Tumors were also fixed in mixed formalin/acetic acid for histological analysis. The xenografts were concomitantly evaluated for their response to 2 chosen treatments. Each tumor was subsequently transplanted into 20 immunodeficient SCID mice for therapeutic assessment.

### 2.3. In Vivo Therapeutic Assessment

**Figure 1:**
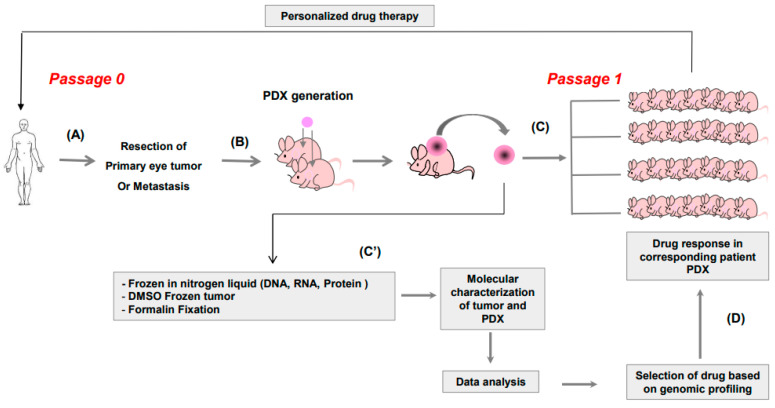
(**A**) Primary tumor in the eye or hepatic metastasis of patients were collected after surgery and (**B**) grafted in interscapular area of 2–5 mice. Once the PDX had grown, each xenograft was collected and (**C**) grafted into 20 mice and (**C’**) also frozen in nitrogen liquid for DNA and RNA extraction. DNA from the initial patient tumor and from the PDX were analyzed by cytoscan HD and fixed in formalin for histology and immunohistochemistry and in DMSO for further regraft, if necessary. Once the target and corresponding drug had been identified, it was administered to the mice (**D**). The most effective drug would be administered to the patient.

For in vivo experiments, everolimus (Certican, Novartis, Basel, Switzerland) was suspended in glucose 5% and administered orally at 2 mg/kg, 5 days/week, for 4 weeks. Dacarbazin (Deticene, Sanofi Aventis, Paris, France) was administered intraperitoneally at 40 mg/kg, for 5 consecutive days. When converted to human equivalents, these doses are similar to those received by patients in the clinic. For in vivo therapeutic studies, 20 female SCID mice (Janvier Labs, Paris, France) were xenografted with a tumor fragment of 20–30 mm^3^ (Figure 1). Mice bearing growing tumors with a volume of 60–200 mm^3^ were randomly assigned to the control or treatment groups (number of animals per group is detailed in the figure legends). Animals with tumor volumes outside this range were excluded. Treatments were started on day 1.

Mice were weighted and tumors measured at least once a week. Xenografted mice were sacrificed after 4 weeks of treatment or when at least one tumor reached a volume of 2500 mm^3^. Relative tumor volumes (RTV) were calculated from the following formula: RTV = (Vx/V1), where Vx is the tumor volume on day x and V1 is the tumor volume at the initiation of therapy (day 1). Growth curves were obtained by plotting the mean values of RTV on the Y axis against time on the X axis, expressed as days after the start of treatment. Antitumor activity was evaluated according to tumor growth inhibition (TGI), calculated according to the formula percent GI = 100 − (RTVt/RTVc × 100), where RTVt is the mean RTV of treated mice and RTVc is the mean RTV of controls, both at a given time point when the antitumor effect was optimal. Fifty percent TGI or more was considered as a meaningful biological effect. Statistical significance of differences observed between the individual RTVs corresponding to the treated mice and control groups was calculated using the two-tailed Mann–Whitney test.

### 2.4. Histopathological Analyses

The morphology of each xenograft was compared with the histological findings of the corresponding patient’s tumor. For light microscopy examination, 4 μm thick AFA-fixed paraffin-embedded sections were stained with H&E Safran.

### 2.5. Establishment of the Tumor Molecular Profile

Frozen tumor (PDX and Patient tumor) samples were used for DNA extraction using the Qiagen^®^ (Venlo, The Netherlands) kit after the evaluation of tumor cell content on a frozen section. Samples containing > 50% of tumor cells were initially considered suitable for DNA extractions and genomic analyses. The extracted tumor DNA was used for mutations and gene copy number analyses as described in [25]. For targeted sequencing, between 10 and 50 ng of DNA was used to prepare indexed paired-end libraries using the Illumina TruSeq Custom Amplicon Low Input (TSCA-li) kit, spanning 6 genes of interest (GNAQ, GNA11, CYSLTR2, EIF1AX, BAP1 and SF3B1) with 1536 amplicons distributed along 21 chromosomes. For CGH, 700 to 1000 ng of tumor DNA and reference DNA were labeled, purified, and cohybridized in equal quantity to NimbleGen arrays or Agilent microarrays. Nimblegen arrays were scanned with a GenePix 4000B scanner using GenePix software V.6.6 (Molecular Devices, San Jose, CA, USA), and data were extracted using NimbleScan software V.2.5. Files produced by Nimblescan software were then analyzed with SignalMap V.1.9. For Agilent, images were acquired with a SureScan microarray scanner using CytoScan software V.2.7, then analyzed with CytoGenomics software V.3.0.2.11.

Selected gene copy number alterations were assessed using Cytoscan HD according to the manufacturer’s protocol (Affymetrix^®^, Santa Clara, CA, USA). Two hundred and fifty ng of genomic DNA was employed to conduct the target preparation and hybridized microarrays. When the amount of available genomic DNA was below 250 ng, a first whole genome amplification (Qiagen, REPLI-g Mini Kit PN:150023) step was implemented to the assay. Negative and positive controls were added to all batches of samples to ensure the quality control of analyses (Affymetrix normal DNA). Hotspot detection was performed by targeted sequencing using the Ion Ampliseq cancer panel V1. The protocols were detailed in [25].

## 3. Results

### 3.1. Study Design

We here aim to evaluate the feasibility of creating avatar PDX models of UM to assess the responses to (targeted) therapy in these models within a timeframe that is compatible with the evolution of this disease in patients. The different steps to obtain a valuable tool for personalized medicine are shown in Figure 1. A total of twenty-nine UM patient samples were obtained after surgery (Figure 1A), among which twenty were from primary UM and nine from liver metastases. Table 1 summarizes the characteristics of the patients. The tumor samples were grafted onto mice (Figure 1B) and once a PDX had grown (passage 0), we fixed one fragment of PDX for histology and froze one fragment to determine the genomic profile of the PDX (Figure 1C’). In parallel, several fragments were frozen to secure the possibility of testing the efficacy of new drugs in the future. The next step was the expansion of the obtained PDXs into 20 new mice (Figure 1C) and, concomitantly, the molecular analysis of PDXs and patients (Figure 1C’). The comparison of PDXs to the corresponding patient’s tumor was used to select compounds for drug screening. Genomic analyses were conducted as described previously [25,26] and results were provided within 2 weeks. The last step is to assess the antitumor efficacy of selected compounds in the mice bearing the xenografts (Figure 1D) to choose the best treatment for the corresponding patient.

### 3.2. Establishment of PDX

We first evaluated the tumor take rate. Tumor take was considered successful when the volume of the PDX tumor reached at least 60 mm^3^. The engraftment was successful for 7/20 (35%) of primary tumors and 6/9 (67%) of metastatic samples (Table 2). There was a tendency for a higher tumor take rate in xenografts obtained from metastases (67%) than those from primary tumors (35%) (*p* = 0.2254). The overall tumor take rate was 45%.

### 3.3. Genomic Risk of the Patient Correlates with Tumor Take Rate in Mice

Copy number alterations of chromosomes 3 and 8 frequently occur in UM and predict metastatic relapse in patients [6]. We determined the genomic risk in patients’ tumor samples and compared this with the tumor take rate in mice (Table 2). High risk (HR) of metastases was defined by the loss of chromosome 3 (chr3) and gain of chromosome 8q in patients [6,27]. The tumor take was around 50% in high-risk patients whose primary tumors displayed both loss of chr3 and gain of 8q (Table 2). In patients with an intermediate risk (IR, either loss of chr3 or gain of chr 8q in the primary tumor), the tumor take rate was 30%. In four patients, chromosome 3 and 8 status was normal. Only one PDX was obtained from these samples, but it stopped growing at passage 1.

In the metastatic patient samples, the take rate was 100% for high-risk tumors, 60% for intermediate-risk, and 0% for low-risk. We thus observed a correlation between tumor take rate and genomic risk as determined by patients’ tumors. One PDX belonging to the high-risk group stopped growing at passage 1. We decided to set aside this tumor for further investigations. The status of chromosome 3 and chromosome 8 and the risk of metastatic relapse for patients whose PDXs were analyzed were reported in Table 2. All PDXs obtained correspond to patients with high or intermediate risk of metastases.

In conclusion, establishing avatar PDX models for high-risk patients was more successful than for low-risk patients. The time needed to obtain the genetic profile and histology of both the patient’s tumor and the corresponding PDX was less than two weeks in our center. This is therefore not a limiting factor for the use of PDX models as avatars for personalized medicine.

Next, we evaluated the time required for the samples that were successfully engrafted to reach 60 mm^3^, which is the volume to be reached to start treatments. This interval was 6 to 11 months for PDXs grown from primary tumor samples and 3 to 13 months for those obtained from metastases (Table 3). Genomic risk did not have an impact on this time interval. Our fastest PDX model (MM270) reached a tumor volume of >60 mm^3^ within three months. A time interval of 2 weeks to obtain the molecular profile was thus convenient for all PDXs.

### 3.4. Conservation of Histological and Genomic Features between Patient’s Tumor and Their PDX

Avatar PDX models can be used to predict response to treatment only if they reliably reflect the patient’s tumor. To determine the extent to which the PDX models maintained the features of the corresponding patient tumors, morphologic and molecular alterations characteristic of UM were studied.

The histomorphology of the PDXs were compared to the corresponding patient tumors by an expert pathologist. As shown in Supplementary Table S1 and Supplementary Figure S1, the histology of patient tumors showed a profile of either epithelioid cells or a mixed type containing both epithelioid and spindle cells. This profile was conserved in all corresponding PDX models, except in the MP257 model, in which epithelioid cells, the more aggressive ones, became dominant in the PDX.

**Figure 2:**
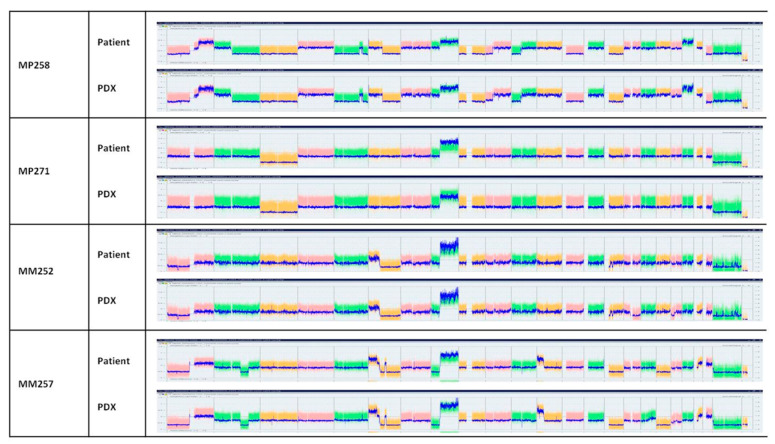
Copy number variation (CNV) of the patients’ samples and their corresponding PDXs in four representative models. Each color represents one chromosome.

Copy number variation (CNV) studies of tumors are shown in Figure 2 and Supplementary Table S1. Figure 2 shows the whole chromosomes of two PDXs from two primary tumors, MP258 and 271, and two PDXs from metastases, MM252 and MM257. Genomic alterations were conserved between patient tumors and their corresponding PDX models. Notably, the two main chromosomes involved in UM, chromosomes 3 and 8, showed the same alterations between the patients and the PDXs (Supplementary Figure S2 and Supplementary Table S1), except for the MP264 and MP262 models, which showed no gain of chromosome 8q in the patient tumors while the PDXs displayed a gain of 8q. However, in these two models, a subclone with a gain of chromosome 8q was detected in the patient’s tumor.

Finally, we characterized the genomic alterations that are recurrently found in UM: mutually exclusive mutations in GNAQ or GNA11, as well as mutations in the BRCA1 associated protein-1 (BAP1) and splicing factor 3b subunit 1 (SF3B1). Patient tumors and their corresponding PDXs showed concordance for all GNAQ, GNA11, BAP1, and SF3B1 mutations (Supplementary Table S1).

In conclusion, PDX models reliably reproduced the major histological and genomic features of patient tumors and could potentially serve as avatar models to assess the best therapeutic options.

### 3.5. Using Avatar Models to Direct Patient Therapy

**Figure 3:**
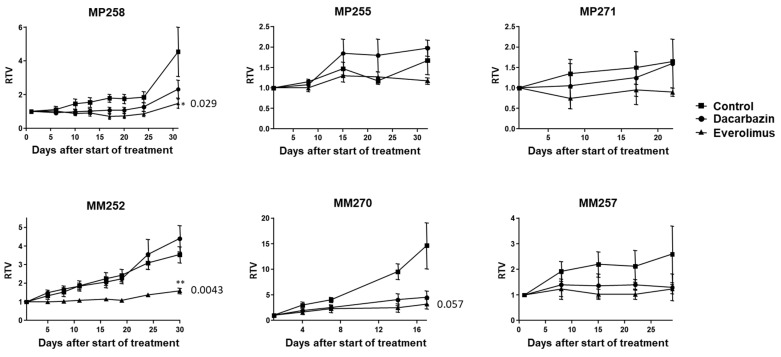
Relative tumor volume (RTV) over time (days) of six PDX models treated with control (square), dacarbazin (circle), or everolimus (triangle). Brackets indicate range, asterisks indicate significant results compared to the control.

Next, we wished to assess if it was feasible to use the PDX models to test several drugs, selected based on the molecular alterations of the tumor, before the relapse of the patient, and thus advise the physician on the treatment to use. For this, the first six tumors obtained at passage 0 were grafted into 20 SCID mice each (=passage 1). The time to reach 60 mm^3^, the volume at which we started drug treatment, was between 3 weeks and 13 months for PDXs obtained from metastatic patient tumors, and between 2.5 and 11 months for PDX obtained from primary tumors. This time interval was shorter in passage 1 compared to passage 0. Once this tumor volume had been reached, the treatments were initiated. For this proof-of-concept study, we chose dacarbazin, an alkylating chemotherapeutic agent approved for the first-line treatment of metastatic UM patients, and everolimus, an mTOR inhibitor that is currently being approved for the treatment of certain types of renal, pancreatic, and breast cancers and that had previously showed some efficacy in UM PDXs [28]. The mice were treated for 4 weeks; relative tumor volume (RTV) was assessed (Figure 3) and tumor growth inhibition (TGI) was calculated. Among the eleven PDXs obtained, six were treated with both drugs: three PDXs obtained from primary tumors (MP255, MP258, and MP271) and three PDXs obtained from metastases (MM252, MM257, and MM270). The tumor growth rate was highly variable: between 30 and 60% for PDXs from primary tumors and between 50 and 95% for PDXs obtained from patient metastases. The number of mice included in each group varied from two to four mice per group for the ones obtained from primary tumors and from three to seven for those obtained from metastases.

Among the three PDXs obtained from primary human tumors, MP258 showed a mild response to dacarbazin (TGI = 49%) and a significant response to everolimus (TGI = 67%, *p* = 0.029). The other two PDXs obtained from primary human tumors (MP255 and MP271) grew slowly and 4 weeks of treatments were insufficient to obtain an antitumor effect (Figure 3).

Among the three PDXs obtained from patient metastases, MM252 showed a significant response to everolimus with TGI = 55% (*p* = 0.0043), while no response was observed to dacarbazin. MM270 showed a high antitumor response to both dacarbazin and everolimus with TGIs = 69% and 78%, respectively (Figure 3). But the number of mice in these groups was too small to reach statistical significance. In the last PDX model, MM257, both drugs tended to stabilize tumor growth, but no significant difference was obtained within 4 weeks, due to slow tumor growth.

### 3.6. Follow-Up of Patient and Corresponding PDX Models

**Figure 4:**
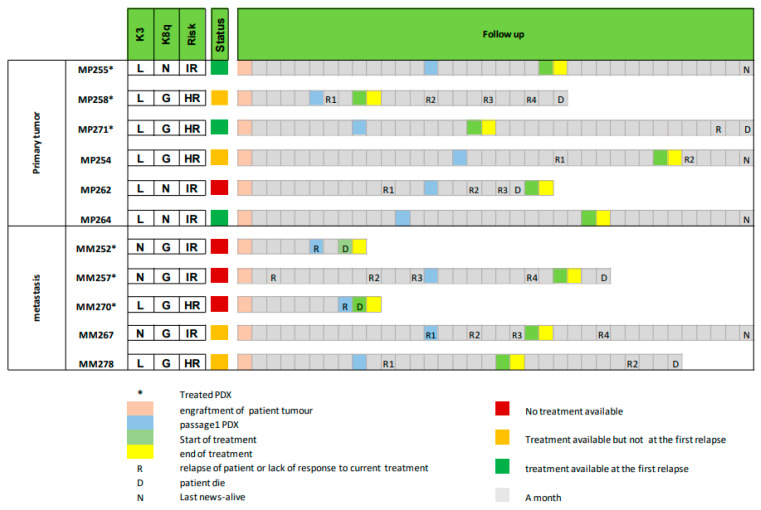
Timeline comparing PDX establishment with the evolution of the corresponding patient. Loss (L), normal (N), or gain (G) of chromosome 3 (K3) and 8q (K8q) as well as genomic risk is indicated for each model (IR: intermediate risk, HR: high risk). Engraftment of tumor (salmon), first passage (blue), start of treatment (light green), and end of treatment (yellow) of the PDXs are indicated in the follow-up. For the patients, each relapse (R), death (D), or latest news (N) are indicated. The column “status” indicates if the PDX avatar could have been used to select a drug for the patient at first relapse (green) or at a subsequent relapse (orange). Red indicates that the patient was deceased before treatment response of PDX was available. Asterisk: PDX models that were treated with dacarbazin and everolimus.

PDX models can be used to choose the best treatment option only if the mice can be treated before the patient relapses. This is a challenge in UM, where relapse often occurs early and prognosis is poor. To define if our PDXs could be used as an avatar model in uveal melanoma, we compared the time required for PDX establishment and treatment with the patients’ follow-up data (Figure 4). The follow-ups started at the moment of the surgery (primary tumor or metastases) which allowed for the grafting of the patient’s tumor into the mice (=Passage 0). In Figure 4, three key time points are indicated for the PDXs: the first passage (blue), the start of treatment (light green), which corresponds to a tumor volume between 60 and 200 mm^3^, and the end of treatment (yellow), for which the duration was set at 4 weeks. The patients were followed for 3 years after the surgery and three events were specified: tumor relapse, latest news, and/or death.

In addition to the six PDXs that were treated with DTIC and everolimus, we added five more PDX models to Figure 4. These PDX models were not treated, but the three indicated key time points (the time of first passage, the time to reach the volume of 60–200 mm^3^, and the end of treatment) were extrapolated from their growth curves during the passages. Among these 11 patients, we determined if the response to treatment in the PDX model was available before the patient relapsed and could thus have been useful for the treatment of the patients. This is illustrated in Figure 4 by a color code. Green corresponds to a PDX that could have helped in selecting the best drug for the patient at the first relapse after surgery (end of treatment (in yellow) occurred before relapse (R)); the orange color corresponds to those cases where the PDX response to treatment was not available at the first relapse but could have been used at subsequent relapses (end of treatment in yellow occurred after relapse 1 but before a subsequent relapse); and finally, the red color indicates those cases in which the patient was deceased before the PDX treatment was completed (end of treatment in yellow occurred after death (D)).

Among the six PDXs obtained from primary tumors, the treatment response of mice could have been used in five patients: for three patients at their first relapse (metastases appeared) (MP255, MP271, and MP264) and for two patients at their second relapse (MP254 and MP258). Only one model, MP262, could not have been used as an avatar model, since the patient was deceased before treatment could be initiated on mice.

Among the five PDXs obtained from metastatic patients, only two PDXs, MM267 and MM278, could have been used to select a drug for their corresponding patients, and not at first relapse.

## 4. Discussion

In this study, we evaluated the feasibility of using PDX models as an avatar to select the best treatment for patients suffering from UM. About half of UM patients develop metastasis, within a time frame that varies from a few months to several years after the diagnosis of the primary disease. Once metastatic, overall survival of UM patients is around 12 months. The timeframe to develop PDX models from metastatic tissue is thus limited, and metastatic tissue is not always available. For this reason, we here evaluated establishing PDX models both from primary tumors and from metastatic tumors.

Using twenty-nine patient samples, among which twenty primary UMs and nine liver metastases, we show that tumor take was better for samples obtained from liver metastases than from primary UM, confirming our previous results in UM [29] as well as observations by others in lung, pancreas, prostate, brain, and colon cancer models ([30,31], reviewed in [32]). This might reflect the fact that metastatic tumors are more frequently high-risk and aggressive tumors, with increased capacity to adapt to a new environment and thus to grow serially in mice. However, once established, we observed that the time for a PDX to grow to a volume of 60 mm^3^ and thus to initiate drug treatment was not significantly different between metastases and primary UM.

Next, we assessed the histology and molecular alterations of the PDX models as compared to the patient samples. Indeed, in our study, the tumors were grafted into the interscapular fat pad, which does not provide the same microenvironment as the liver. However, it is a well vascularized environment, and it facilitates the follow-up of tumor growth. Orthotopic engraftment into the liver or the eye was not an option here, because it is an invasive procedure requiring expensive imaging and repeated anesthesia, with a high risk of infection or death of the animal and thus loss of the tumor sample. We have previously shown that PDXs grafted into the interscapular fat pad remain very similar to the human tumor in terms of histology and molecular biology [29,31,32]. Thanks to the organization that was put in place at Institut Curie for the SHIVA01 trial [25,26], alongside genomics platforms, the time to analyze the histology and the molecular alterations of PDX and patient samples was as short as 2 weeks. In concordance with our previous results, we show a very high similarity between the patient samples and their corresponding PDX models at the level of histology, copy number alterations and genomic variations, both for models derived from primary tumors and from metastases. This confirms our previous observations [29,33] and validates the reliability and clinical value of our UM models. Interestingly, two PDX models showed a gain of 8q that was present as a subclone in the primary patient tumor. This genomic alteration is associated with increased metastatic risk [6]. We hypothesize that this was an aggressive subclone of the primary tumor that had a growth advantage upon engraftment in the mice [34,35]. Moreover, it might have been the clone leading to metastasis in the patient, as has been observed previously [35,36,37], in which case the PDX model would have been extremely helpful.

Finally, we evaluated the feasibility of obtaining clinically relevant data from PDX models within a time frame that is compatible with the evolution of the patients’ disease. For this feasibility study, we here decided to treat the mice with two standardized treatments: dacarbazin, an alkylating chemotherapeutic agent that is approved for first-line treatment of metastatic UM, and everolimus, an mTOR inhibitor that had previously showed some efficacy in UM PDX models [28,38]. We fixed our treatment duration at 4 weeks. We show that, for the PDXs derived from the primary tumors, drug response could have been available at the first relapse after the surgery for three out of six patients. For two other patients, the chosen treatment could have been used after the second relapse. The remaining patient showed rapid disease evolution and passed away before the PDX could be treated. In the metastatic patients, none of the six PDXs could have been informative at the first relapse, although two out of five could have been used at subsequent relapses. In conclusion, PDXs from primary tumors could have been used as avatar models in 50% of the cases if we consider only the first relapse and 85% if we consider all relapses, against 0% and 40%, respectively, for PDXs derived from metastases. We noticed that 4 weeks of treatment was too short in most mice to obtain a frank tumor response, due to the slow growth of the PDX models, and that 6 or 8 weeks of treatment would probably have been preferable. Importantly, in our models, an extension of the treatment length by two or four weeks would not have modified our conclusions in terms of feasibility and utility for the patient for PDXs obtained from primary tumors.

In this study, we demonstrate for the first time the technical feasibility of developing a PDX avatar model from UM primary tumor samples, and to test multiple drugs in parallel in order to select the best drug to treat the patient. The clinical interest of PDX models derived from primary tumors has been questioned in the literature because genomic evolution that differed from the one observed in humans has been detected in mice [39]. However, UM is a very genetically stable disease [24,40], with few genomic evolutions between the primary tumor and the metastases. This exceptional genetic stability of UM over time, shown by us and others [24,40], allows us to envisage the use of primary tumors to explore treatment options for patients once they become metastatic. A similar avatar approach is currently under clinical evaluation in indications such as breast cancer (NCT05464082), non-small-cell lung cancer (NCT03134456), mantle cell lymphoma (NCT03219047-2018-2023), and pancreatic cancer (NCT04373928), and would be worth testing in UM as well. The only limitation so far is that the mice used in our study are immunodeficient and can therefore not be used to test immunotherapies such as the recently approved tebentafusp [41]. However, the development of humanized mouse models may be able to overcome this limitation in the near future [42].

The fraction of patients that could benefit from the avatar approach depends on the tumor take rate and the time required to grow and treat the mouse model. Our results show a take rate of six out of sixteen (38%) among primary UM tumors displaying a high or intermediate genomic risk of relapse. Among these six established models, response to treatment was available before the patient relapsed in five out of six models (83%). These findings should be confirmed in more patients but suggest that the avatar model could be useful in 31% of primary UM patients with high or intermediate risk of relapse. This patient population has the highest need for new personalized treatment options and would be the population of choice to evaluate the UM avatar approach prospectively in the clinical setting. In conclusion, one out of three UM patients with high or intermediate risk of relapse could benefit from avatar models to select in advance the best treatment option to be used at the moment of relapse. This would represent an opportunity in this disease where treatment options are limited.

## Figures and Tables

**Table 1:** Patient characteristics.

Characteristics	Nb of Patients
Patients	29 *
Sex	
Male	12
Female	17
Age (year range)	
Male	31–81
Female	31–83
Tumors	
Primary	20
Metastasis	9

* Among 30 tumors collected, one patient sample was not a tumor.

**Table 2:** Tumor take rate and genomic risk.

Grafted Patient Samples	Tumor Take Rate (TTR)	Origin	Tumor Take (TTR)	Genomic Classification	Tumor Take Rate (TTR)
29	13/29 45%	Primary tumor (20 grafted tumors)	7/20 (35%)	High Risk	3/6 (50%)
				Intermediate	3/10 (30%)
				Low Risk	¼ * (25%)
		Liver metastasis (9 grafted tumors)	6/9 (67%)	High Risk	3/3 * (100%)
				Intermediate	3/5 (60%)
				Low Risk	0/1 (0%)

TTR: Tumor take rate; * 1 tumor stopped growing at passage 1.

**Table 3:** Time needed to obtain the PDX model.

	PDX	PDX Passage 0 ^$^	Chr 3	Chr 8q	Risk of Metastases *	Time to Reach Volume > 60 mm^3^ (Months) ^£^
**PDX from** **Patients’ metastasis**	MM252	24/06/13	N	G	IR	3
	MM257	23/09/13	N	G	IR	10
	MM270	17/02/14	L	G	HR	7
	MM267	03/02/14	N	G	IR	13
	MM278	10/04/14	L	G	HR	4
	MM287	25/08/14	L	G	HR	12 ^@^
**PDX from** **Patients’primary tumor**	MP255	03/09/13	L	N	IR	7
	MP258	25/09/13	L	N	IR	6
	MP271	19/02/14	L	G	HR	6
	MP254	30/08/13	L	G	HR	9
	MP262	17/12/13	L	N	IR	8
	MP264	27/01/14	L	N	IR	11
	MP266	29/01/14	N	N	LR	14 ^@^

^$^ Graft PDX = date of surgery = Day 1; * Cassoux et al., BJO; 2014; ^£^ Theoretical time (months) from surgery except for underlined PDX; L: Loss; N: normal; G: gain. @: tumors stopped growing at passage 1.

## Data Availability

The data presented in this study are available on request from the corresponding author. The data are not publicly available due to patient privacy and GDPR regulations.

## Supplementary Materials

The following supporting information can be downloaded at: https://www.mdpi.com/article/10.3390/curroncol30100657/s1, Table S1: Comparison of patient samples and PDX models in terms of histology, copy number variation (CNV) and mutation status; Figure S1: Histological comparison of morphology of patient samples and their corresponding PDXs in each of the established models. Left panel: models established from primary tumors; right panel: model established from metastases; Figure S2: Zoom in on copy number variation (CNV) of chromosome 3 (left) and 8 (right) in patient samples and their corresponding PDXs in four representative models.
