# Supplementary material for: Patient Derived Xenografts (PDX) Models as an Avatar to Assess Personalized Therapy Options in Uveal Melanoma: A Feasibility Study
Source: Curr Oncol. 2023 Oct 11;30(10):9090–103. doi: 10.3390/curroncol30100657 (PMC10604955; doi:10.3390/curroncol30100657)

Supplementary Table S1. Comparison of Patient and PDX

Models		HISTOLOGY	K3	K8	Method	*GNAQ*	*GNA11*	*SF3B1*	*BAP1*
MP254	PATIENT	M	L	G	PUMA	mut	wt	wt	mut
	PDX	M	L	G	PUMA	mut	wt	wt	mut
MP255	PATIENT	M	L	N	PUMA	mut	wt	wt	wt
	PDX	M	L	N	PUMA	mut	wt	wt	wt
MP258	PATIENT	M	L	G	TSCA	wt	mut	wt	mut
	PDX	M	L	G	PUMA	wt	mut	wt	mut
MP262	PATIENT	M	L	N$	PUMA	wt	mut	wt	mut
	PDX	M	L	G	PUMA	wt	mut	wt	mut
MP264	PATIENT	M	L	N$	PUMA	wt	mut	wt	mut
	PDX	M	L	G	PUMA	wt	mut	wt	mut
MP271	PATIENT	M	L	G	SANGER	wt	mut	ND	ND
	PDX	M	L	G	PUMA	wt	mut	wt	wt
MM252	PATIENT	M	isodisomy	G	PUMA	mut	wt	wt	wt
	PDX	M	isodisomy	G	PUMA	mut	wt	wt	wt
MM257	PATIENT*	M	N	G	PUMA	mut	wt	mut	wt
	PDX	E	N	G	PUMA	mut	wt	mut	wt
MM270	PATIENT	M	isodisomy	G	PUMA	mut	wt	wt	wt
	PDX	E	L	G	PUMA	mut	wt	wt	wt
MM267	PATIENT	M	N	G	PUMA	wt	mut	mut	wt
	PDX	M	N	G	PUMA	wt	mut	mut	wt
MM278	PATIENT	E	L	G	PUMA	mut	wt	wt	mut
	PDX	E	L	G	PUMA	mut	wt	wt	mut

K: chromosome; M : Mixt means epithelioid + spindle cells ; E: Epithelioid cells ; G: gain ; wt: wild type gene; mut: mutated gene ; ND: not done ; * Primary tumor analysis ; $Subclonal 8q gain.

Supplementary Figure S1. Morphological comparison of patient and corresponding PDX

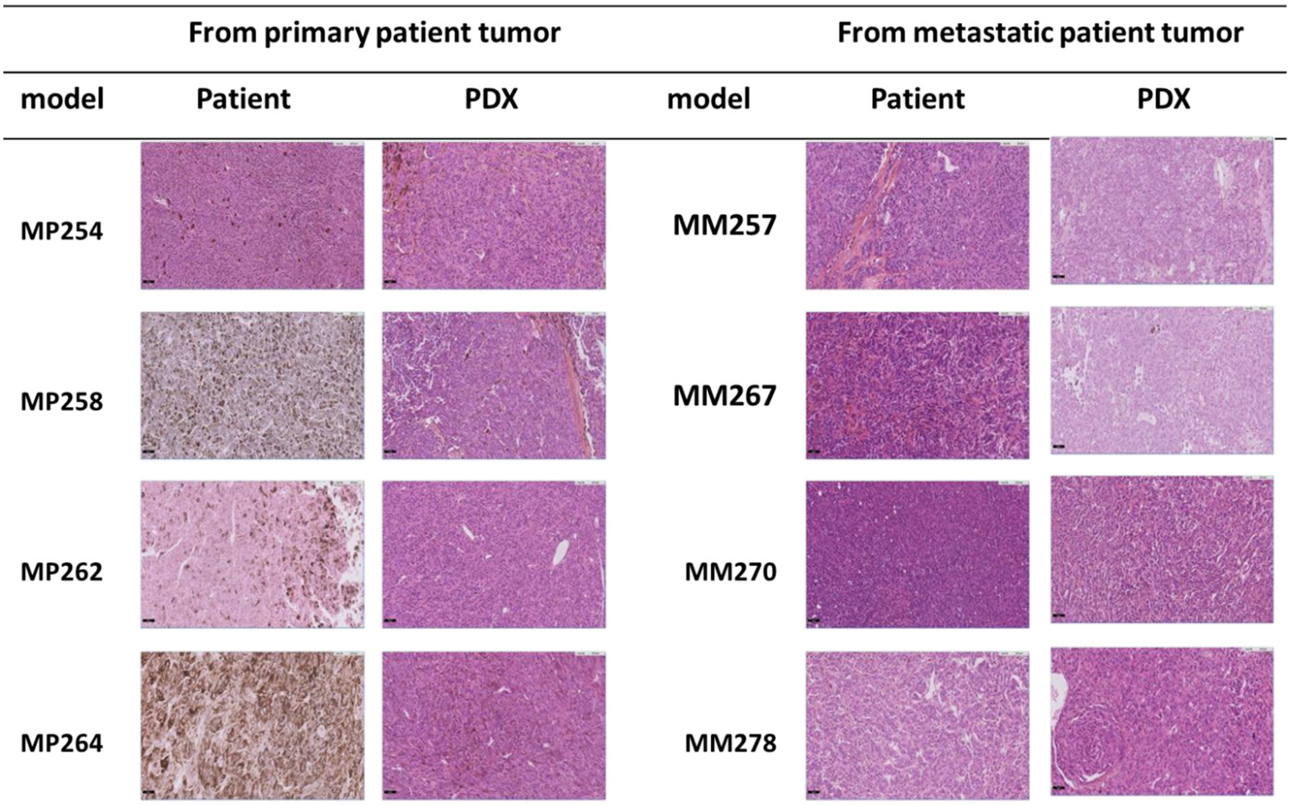

Supplementary Figure S2. Copy number variation of chromosomes 3 and 8: comparison between patients and their corresponding PDX

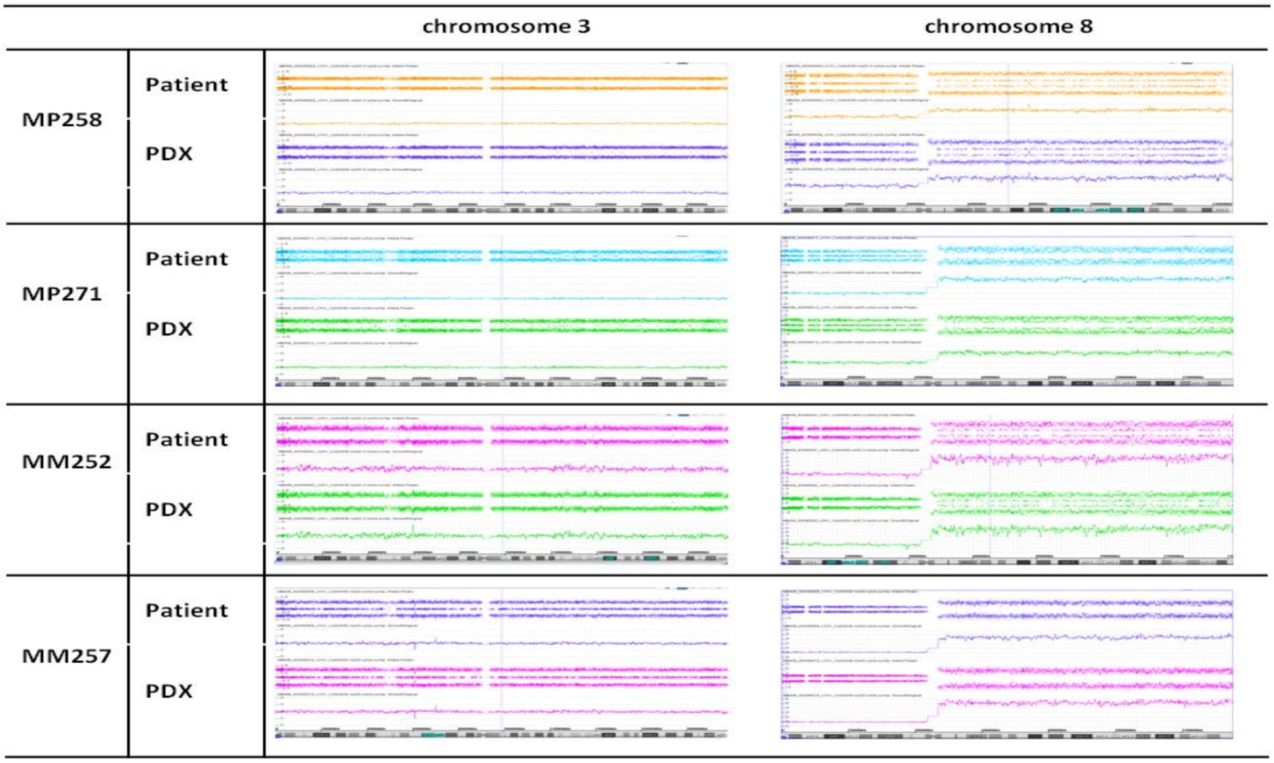

Supplement: Supplementary file 1 [file curroncol-30-00657-s001.zip › curroncol-2567629-supplementary.pdf]
